# Identification and Assessment of lncRNAs and mRNAs in PM2.5-Induced Hepatic Steatosis

**DOI:** 10.3390/ijms26062808

**Published:** 2025-03-20

**Authors:** Peixuan Tian, Hui Xia, Xinbao Li, Ying Wang, Bihuan Hu, Yu Yang, Guiju Sun, Jing Sui

**Affiliations:** 1 Key Laboratory of Environmental Medicine and Engineering of Ministry of Education, Department of Nutrition and Food Hygiene, School of Public Health, Southeast University, Nanjing 210009, China; 220244286@seu.edu.cn (P.T.); huixia@seu.edu.cn (H.X.); gjsun@seu.edu.cn (G.S.); 2 Research Institute for Environment and Health, Nanjing University of Information Science and Technology, Nanjing 210044, China

**Keywords:** PM2.5, non-alcoholic fatty liver disease, long non-coding RNAs, weighted gene co-expression network analysis

## Abstract

Research indicates that fine particulate matter (PM2.5) exposure is associated with the onset of non-alcoholic fatty liver disease (NAFLD), the most prevalent chronic liver disorder. However, the underlying pathogenesis mechanisms remain to be fully understood. Our study investigated the hub long non-coding RNAs (lncRNAs) and messenger RNAs (mRNAs) associated with hepatic steatosis caused by PM2.5 exposure and their pathological mechanisms. The analysis of gene profiles in the GSE186900 dataset from the Gene Expression Omnibus (GEO) enabled the identification of 38 differentially expressed lncRNAs and 1945 mRNAs. To explore further, a co-expression network was established utilizing weighted gene co-expression network analysis (WGCNA). Moreover, Gene Ontology (GO) and Kyoto Encyclopedia of Genes and Genomes (KEGG) databases were utilized for functional enrichment analysis. Our analysis identified specific modules, particularly the blue and turquoise modules, which showed a strong correlation with NAFLD. Through functional enrichment analysis, we identified several lncRNAs (including *Gm15446*, *Tmem181b-ps*, *Adh6-ps1*, *Gm5848*, *Zfp141*, *Rmrp*, and *Rb1*) which may be involved in modulating NAFLD, multiple metabolic pathways, inflammation, cell senescence, apoptosis, oxidative stress, and various signaling pathways. The hub lncRNAs identified in our study provide novel biomarkers and potential targets for the diagnosis and treatment of NAFLD.

## 1. Introduction

Non-alcoholic fatty liver disease (NAFLD) encompasses non-alcoholic fatty liver (NAFL), nonalcoholic steatohepatitis (NASH), and progressive fibrosis leading to cirrhosis [1]. It is the predominant chronic liver disease and represents the hepatic manifestation of metabolic syndrome. NAFLD can progressively advance to extrahepatic cancer, hepatocellular carcinoma (HCC), cirrhosis, or cardiovascular disease, thereby raising both all-cause and cause-specific mortality rates [2]. The number of NAFLD cases is increasing, with studies in China indicating that the prevalence of NAFLD has increased threefold over the past decade [3].

Ambient air pollution has become a significant global threat, raising considerable concern worldwide [4]. Air pollutants include particulate matter (PM) and gaseous pollutants. Fine particulate matter (PM2.5) refers to airborne particles with an aerodynamic diameter (AED) below 2.5 μm in the surrounding environment. PM2.5 stems from natural and anthropogenic sources, with industrial emissions and vehicle exhaust being dominant contributors in urban regions [5]. Recently, based on evidence accumulated over the past 15 years, the World Health Organization (WHO) released updated air quality guidelines, establishing the PM2.5 benchmark at 5 μg/m^3^ [6].

A growing body of research indicates that air pollution could elevate the risk of dyslipidemia related to NAFLD. Moreover, studies have shown that once inhaled, PM2.5 particles can deposit in the pulmonary alveoli, traverse the air–blood barrier through pulmonary ventilation, enter the liver via blood circulation, and participate in hepatic metabolism, thereby leading to liver damage characterized by steatosis. This indicates that the liver is a significant target organ for PM2.5 exposure [7]. A recent meta-analysis and systematic review [8] of 14 studies revealed that PM2.5-generated air pollution is positively correlated with the prevalence of NAFLD and its associated cirrhosis, with an odds ratio (OR) of 1.33 (95% CI: 1.25, 1.42). Notably, the heightened risk of NAFLD and its associated cirrhosis due to PM2.5 exposure in developing countries, with an OR of 1.41 (95% CI: 1.29, 1.53), is noticeably higher than that observed in developed countries. A Chinese cohort study examining population dynamics identified a robust association between PM2.5 concentrations and increased NAFLD risk across all models. In contrast to individuals in the lowest reference group, those in the highest quartile of PM2.5 exposure exhibited hazard ratios (HRs) for NAFLD of 2.70 in the unadjusted model and 2.04 in the fully adjusted model. Additionally, each 10 μg/m^3^ increase in PM2.5 concentration was correlated with a higher NAFLD incidence risk in both unadjusted and adjusted analyses (*p* < 0.05) [9]. A prospective cohort study from the UK indicated that extended exposure to PM2.5 and NO_2_ heightened the risk of NAFLD, partly by disrupting circulating proteins involved in immune and inflammatory pathways [10]. In animal studies, Xu et al. [11] confirmed that exposure to PM2.5 in mice resulted in increased insulin resistance, peripheral inflammation, and dysregulation of metabolic pathways. This research further suggested that inhalation of PM2.5 induces oxidative stress and inflammatory responses, leading to impaired hepatic function and promotion of lipid accumulation in the liver. Lin et al. [12] examined liver transcriptomic alterations in the ob/ob mice following concentrated PM2.5 exposure and indicated that exposure to PM2.5 could affect insulin sensitivity, glucose metabolism, and fatty liver lipid processing in obesity, potentially contributing to the onset and development of PM2.5-induced liver disorders, including NAFLD and HCC. This evidence indicates that PM2.5 exposure could significantly contribute to the risk of NAFLD. However, the precise pathogenesis mechanisms through which PM2.5 influences NAFLD remain incompletely understood.

Hub long non-coding RNAs (lncRNAs) are non-coding RNA transcripts longer than 200 nucleotides. In the cytoplasm, lncRNAs interact with mRNAs to regulate splicing, maturation, transport, stability, and translation [13]. Numerous studies highlight the critical biological functions of hub lncRNAs in diverse diseases [14,15,16]. Furthermore, research has indicated that lncRNAs play essential regulatory roles in the pathophysiological mechanisms underlying NAFLD [17]. A systematic review [18] of lncRNAs and circRNAs in patients with NAFLD has shown that sixteen studies (including 22 lncRNAs) identified differentially expressed lncRNAs in individuals with NAFLD. Among these, 15 lncRNAs showed increased expression (mean fold-change = 3.26, range: 1.32, 8.30), while 6 lncRNAs exhibited decreased expression (mean fold-change = 0.43, range: 0.13, 0.74). Moreover, lncRNAs significantly influence critical functions, including the regulation of oxidative stress, glucose and lipid metabolism, inflammatory, and immune responses, thereby profoundly affecting various metabolic processes within the liver. Consequently, these lncRNA-induced metabolic dysfunctions trigger the onset and progression of NAFLD. Additionally, previous studies indicated that predicting the roles of lncRNAs through their mRNA-related co-expression networks can significantly enhance the understanding and future investigation into NAFLD [19,20].

Weighted gene co-expression network analysis (WGCNA) is a computational approach for identifying co-expression modules in gene expression datasets [21]. WGCNA detects connections within strongly co-expressed modules and reveals dynamics of gene interactions, including hub lncRNAs and mRNAs [22,23,24,25]. To elucidate the hub lncRNAs and mRNAs involved in the disease mechanisms of PM2.5-induced liver steatosis, we analyzed data using the Gene Expression Omnibus (GEO) (GSE186900). WGCNA was utilized to identify co-expressed lncRNAs and mRNAs modules and interactions. After constructing the lncRNA-mRNA-nets and lncRNA-mRNA-pathway-nets, we investigated the regulatory functions of lncRNAs in modulating mRNAs associated with PM2.5-induced NAFLD, thereby enhancing our insight into lncRNA biological roles.

## 2. Results

### 2.1. Global Differentially Expressed Gene Patterns in Liver Tissue

**Figure 1:**
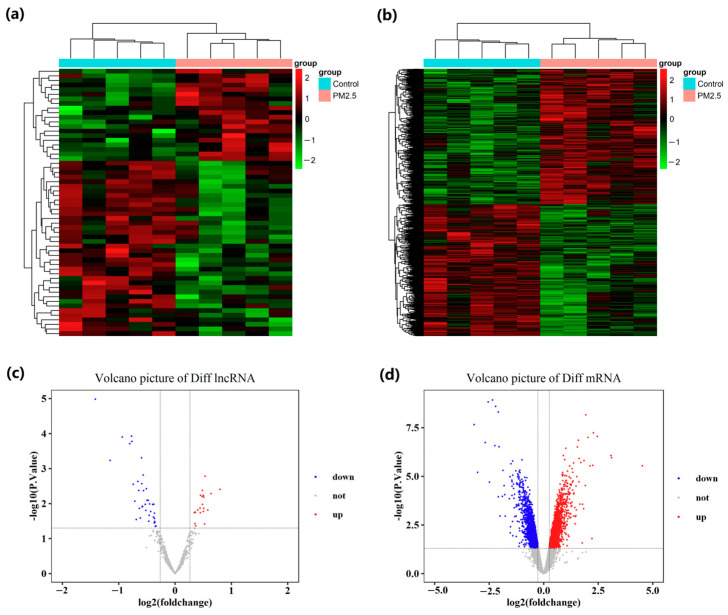
Differential expression analysis of lncRNAs and mRNAs in PM2.5 exposure conditions. (**a**,**c**) lncRNAs; (**b**,**d**) mRNAs.

Among all 4003 differentially expressed genes (DEGs) identified using the set criteria (*p* < 0.05 and fold change (FC) > 1.2), there were 58 lncRNAs and 3945 mRNAs. Specifically, 2020 genes were up-regulated (20 lncRNAs and 2000 mRNAs), while 1983 genes were down-regulated (38 lncRNAs and 1945 mRNAs). The cluster heat map (Figure 1a,b) and the volcano plot (Figure 1c,d) illustrated comparative levels among differentially expressed lncRNAs and mRNAs following PM2.5 exposure.

### 2.2. GO and KEGG Pathway Enrichment Analysis of DEGs

**Figure 2:**
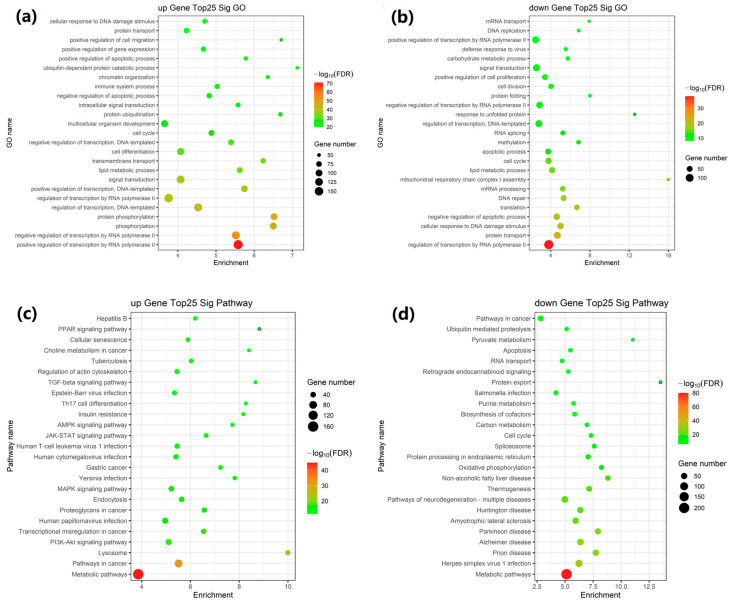
Functional enrichment analysis of DEGs. (**a**,**b**) GO enrichment analysis of up-regulated and down-regulated mRNAs; (**c**,**d**) KEGG pathway enrichment analysis.

The Gene Ontology (GO) enrichment analysis indicated that DEGs were primarily enriched in the cell cycle, regulation of transcription, lipid metabolic process, signal transduction, protein transport, RNA polymerase II, phosphorylation, transmembrane transport, and negative regulation of the apoptotic process (Figure 2a,b).

The pathway enrichment analysis using the Kyoto Encyclopedia of Genes and Genomes (KEGG) database indicated that these DEGs were primarily associated with AMPK, PI3K-Akt, PPAR, MAPK, and JAK-STAT signaling pathways, pathways in cancer, apoptosis, metabolic pathways, pathways of neurodegeneration-multiple diseases, oxidative phosphorylation, insulin resistance, herpes simplex virus 1 infection, NAFLD, and lysosomes (Figure 2c,d).

### 2.3. Construction of Weighted Co-Expression Network and Identification of Crucial Modules

**Figure 3:**
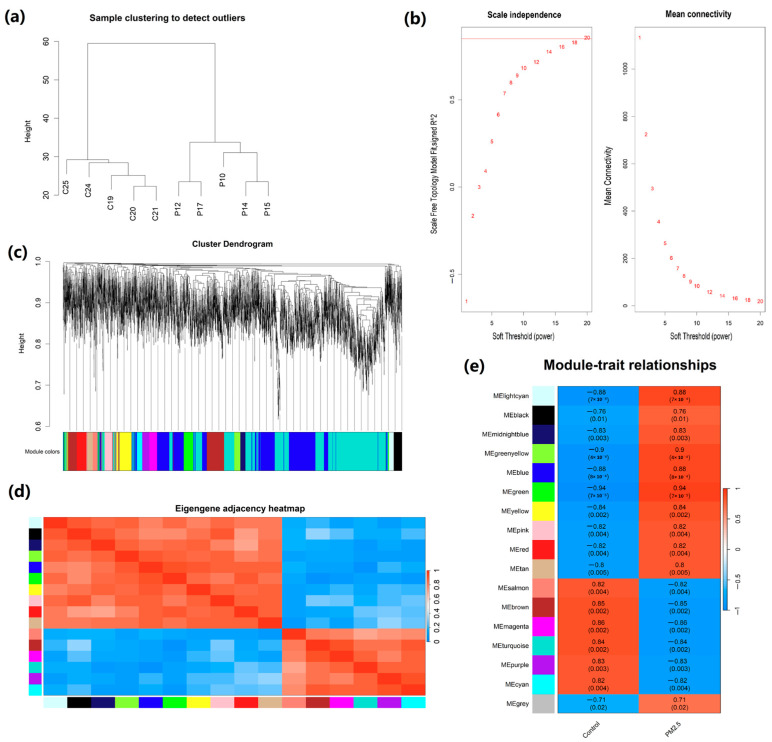
WGCNA was used to identify co-expression modules among differentially expressed genes. (**a**) No outlier samples were identified based on the cut-off height; (**b**) the soft threshold power determination; (**c**) clustering dendrograms of WGCNA (derived from the dynamic tree cutting method the colorful bands facilitate direct visual module allocation contrasts); (**d**) heatmap of multiple gene modules; (**e**) correlation between modular genes and NAFLD (each cell contains the correlation coefficient and the *p*-value).

Cluster analysis of the GSE186900 dataset revealed 58 lncRNAs and 3945 mRNAs from 10 samples (Figure 1). To comprehensively profile lncRNA and mRNA expression, we performed hierarchical clustering to identify potential outlier samples. The results showed no presence of outlier samples (Figure 3a). The analysis of scale independence and average connectivity determined a soft threshold power (β) of 20 (Figure 3b). Subsequently, hierarchical clustering dendrograms were performed to identify diverse modules based on the β value (Figure 3c). Seventeen modules, including blue, turquoise, brown, green, yellow, pink, red, and purple, were identified through dynamic shearing. The number of lncRNAs and mRNAs within these modules is displayed in Table 1. The connections among modules were illustrated through an eigengene adjacency heatmap (Figure 3d).

### 2.4. Correlation Analysis Between Modules and Traits

The connection between co-expression modules and exposure to PM2.5 is illustrated in Figure 3e. We identified that the top three modules correlated with PM2.5 exposure are the green module (correlation coefficient = 0.94, *p* = 7 × 10^−5^), greenyellow module (correlation coefficient = 0.90, *p* = 4 × 10^−4^), and blue module (correlation coefficient = 0.88, *p* = 8 × 10^−4^). By considering both the correlation between modular genes and PM2.5 exposure (Figure 3e) and the number of lncRNAs and mRNAs across all modules (Table 1), we selected lncRNAs and mRNAs from blue, turquoise, brown, green, and yellow modules for subsequent network regulation analysis.

### 2.5. GO and KEGG Pathway Enrichment Analysis of Network Modules

**Figure 4:**
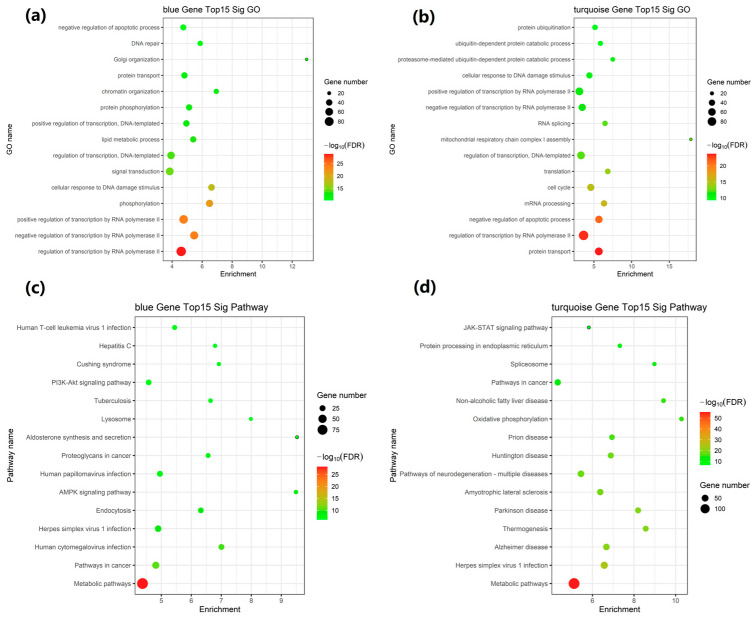
Functional enrichment evaluation in the blue and turquoise modules. (**a**,**b**) GO enrichment analysis of mRNAs within the blue and turquoise modules; (**c**,**d**) KEGG Pathway enrichment analysis.

We conducted GO and KEGG pathway enrichment analyses to evaluate functional gene similarity and investigate the biological relevance of the network modules and subsequently performed these analyses on the mRNAs within the blue, turquoise, brown, green, and yellow modules. The results demonstrated that the top 15 GO terms were significantly enriched in processes such as lipid metabolic process, signal transduction, the regulation of transcription by RNA polymerase II, phosphorylation, negative regulation of apoptotic process, protein transport, cell cycle, regulation of transcription, RNA splicing, and translation. These findings for the blue and turquoise modules are illustrated in Figure 4a,b and Table 2.

KEGG pathway analysis revealed that differential mRNA expression was significantly associated with pathways in cancer, metabolic pathways, pathways of neurodegeneration-multiple diseases (including Alzheimer’s disease, Parkinson’s disease, and Huntington’s disease), herpes simplex virus 1 infection, NAFLD, AMPK, PI3K-Akt and JAK-STAT signaling pathways, and oxidative phosphorylation lysosomes (Figure 4c,d and Table 3). Additionally, GO enrichment analyses and the KEGG pathway analyses for the brown, green, and yellow modules are presented in Supplementary Figure S1. Overall, the findings strongly associate the genes in these modules with signaling and metabolic pathways.

### 2.6. Construction of lncRNA-mRNA-Nets

**Figure 5:**
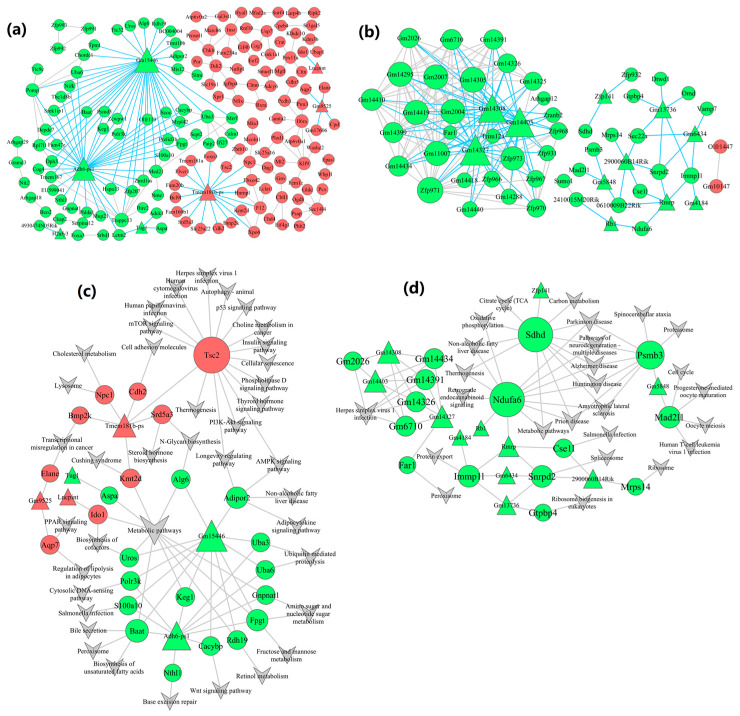
lncRNA-mRNA-nets and lncRNA-mRNA-pathway-nets of hub genes. (**a**,**b**) lncRNA-mRNA-nets and lncRNA-mRNA-pathway-nets of the blue module; (**c**,**d**) the turquoise module. (Triangles represent lncRNAs, circles represent mRNAs, as well as red and green graphics indicate up-regulated and down-regulated genes. The size reflects the regulatory ability.)

lncRNA-mRNA-nets were constructed to elucidate the functional roles of lncRNAs within essential modules (Figure 5a,b). A total of 7 lncRNAs and 156 mRNAs were revealed as hub genes in the blue module (Figure 5a), while 11 lncRNAs and 47 mRNAs hubs were revealed in the turquoise module (Figure 5b). We observed inter-regulatory interactions between lncRNAs and mRNAs. Specifically, a single lncRNA was co-expressed with various mRNAs, while several lncRNAs were co-expressed with individual mRNAs, revealing a complex regulatory network within the lncRNA-mRNA-nets. Multiple lncRNAs exhibited strong associations with key mRNAs within these co-expression networks. Consequently, the potential functions of these lncRNAs can be inferred based on the established functions of their associated mRNAs.

### 2.7. Construction of lncRNA-mRNA-Pathway-Nets

We compared the notably distinct pathways and the lncRNA-mRNA-pathway-nets to derive the lncRNA-mRNA-pathway-nets (Figure 5c,d) and elucidate the possible mechanisms by which lncRNA influences signaling pathways. The blue module within this network comprised 6 lncRNAs and 24 mRNAs (Figure 5c), while the turquoise module included 11 lncRNAs and 15 mRNAs (Figure 5d). The lncRNA-mRNA-nets and lncRNA-mRNA-pathway-nets of hub genes within the brown, green, and yellow modules are shown in Supplementary Figure S2.

In the blue module, *Gm15446* (a lncRNA) was associated with twelve mRNAs (including *Adipor2*, *Keg1*, *Baat*, *Polr3k*, and *Uros*) and enriched in NAFLD, metabolic pathways, adipocytokine signaling pathways, biosynthesis of unsaturated fatty acids, Wnt signaling pathways, metabolic pathways, amino sugar, nucleotide sugar, fructose, mannose, as well as retinol metabolism and bile secretion. *Adh6-ps1* (a lncRNA) was related to ten mRNAs (including *Baat*, *Gnpnat1*, *Keg1*, *Uba6*, and *Uba3*) and abundant in the biosynthesis of unsaturated fatty acids, Wnt signaling pathways, peroxisome, and base excision repair. And *Adh6-ps1*, in conjunction with *Gm15446*, regulates the expression of eight mRNAs (*Baat*, *Cacybp*, *Fpgt*, *Keg1*, *Polr3k*, *S100a10*, *Uba6*, and *Uba3*). *Tmem181b-ps* (a lncRNA) was related to six mRNAs (*Tsc2*, *Srd5a3*, *Cdh2*, *Npc1*, *Bmp2k*, and *Kmt2d*) and enriched in autophagy-animal, cellular senescence, metabolic pathways, mTOR, AMPK, and p53 signaling pathways, insulin signaling pathways, cholesterol metabolism, lysosomes, human papillomavirus infection, and herpes simplex virus 1 infection.

In the turquoise module, *Zfp141* (a lncRNA) was associated with one mRNA (*Sdhd*) and abundant in NAFLD, metabolic pathways, pathways of neurodegeneration-multiple diseases (including Alzheimer’s disease, Parkinson’s disease, and Huntington’s disease), carbon metabolism, and oxidative phosphorylation. *Gm5848* (a lncRNA) was connected with two mRNAs (*Psmb3* and *Mad2l1*) and enriched in pathways of neurodegeneration-multiple diseases, cell cycle, proteasome, and human T-cell leukemia virus 1 infection. *Rmrp* (a lncRNA) was associated with four mRNAs (*Ndufa6*, *Cse1l*, *Immp11*, and *Snrpd2*) and enriched in metabolic pathways, pathways of neurodegeneration-multiple diseases, NAFLD, cell cycle, protein export, and Spliceosome. *Rb1* (a lncRNA) was related to one mRNA (*Ndufa6*) and co-regulates *Ndufa6* in conjunction with *Rmrp*.

### 2.8. Construction of Protein–Protein Interaction Network

**Figure 6:**
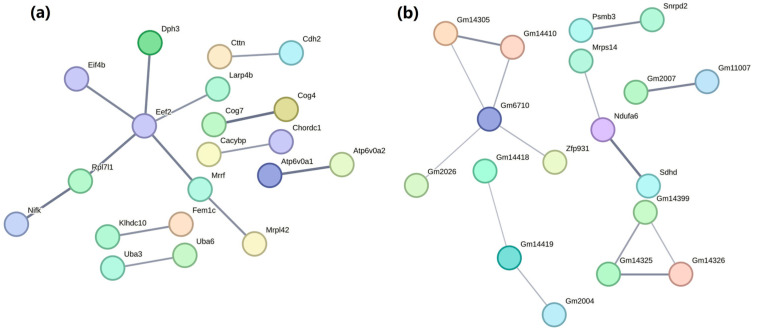
Protein–protein interaction (PPI) networks of the blue (**a**) and the turquoise (**b**) modules. (The thickness of the line represents edge confidence, with thicker lines indicating higher confidence levels).

We utilized the STRING database to create PPI networks, analyzing hub gene interactions in each module (Figure 6). Using PPI analysis, we identified 20 crucial genes in the PPI network of the blue module, as illustrated in Figure 6a. Meanwhile, 18 hub genes were identified in the turquoise module, as shown in Figure 6b.

## 3. Discussion

Numerous studies have established a significant link between PM2.5 exposure and NAFLD, confirming variations in gene expression between NAFLD patients and healthy individuals [26,27]. Moreover, previous studies have demonstrated that exposure to PM2.5 leads to increased hepatic lipid accumulation and have indicated that air pollution is associated with metabolic abnormalities related to NAFLD [28,29]. Nevertheless, the precise mechanisms underlying PM2.5-induced NAFLD remain incompletely understood.

NAFLD is caused by the excessive accumulation and degeneration of lipids in the liver. In this study, we found that PM2.5 exposure induces abnormal lipid metabolism and its associated pathways, thereby significantly influencing the progression of NAFLD. It provides novel evidence establishing a link between air pollution and both the onset and progression of NAFLD. By utilizing the GEO database (GSE186900), we identified 58 lncRNAs and 3945 mRNAs. Following WGCNA analysis, lncRNAs and mRNAs were grouped into 17 functionally distinct modules. Among these, five modules (blue, turquoise, brown, green, and yellow modules) displayed strong correlations with NAFLD. To examine the possible biological roles of key genes in NAFLD, we conducted a functional enrichment analysis. Furthermore, we constructed lncRNA-mRNA-nets and lncRNA-mRNA-pathway-nets to pinpoint critical regulatory relationships. Our findings indicated that lncRNA-mRNA interactions are associated with cell senescence and apoptosis, lipid metabolism, oxidative stress, inflammation, metabolic pathways, as well as p53, AMPK, WNT, PI3K-AKT, and mTOR signaling pathways. It was proposed that PM2.5 exposure may contribute to the development of NAFLD by disrupting multiple signaling pathways and dysregulating metabolic processes in the liver.

WGCNA utilizes gene expression profiles to detect modules and correlate them with phenotypic traits [30]. The key genes discovered through WGCNA are vital for regulating biological functions [31,32,33,34,35]. Therefore, in the WGCNA analysis conducted in our study, we concentrated on the key genes within the blue and turquoise modules. Several key lncRNAs were identified as playing critical roles in regulating essential biological processes among these modules. Our findings indicate that the synergistic interactions among multiple genes contribute to cell senescence, apoptosis, oxidative stress, inflammation, and dysregulation of metabolic pathways in the liver, thereby promoting the development of NAFLD.

LncRNAs are capable of interacting with RNA, proteins, and even DNA. As a result, lncRNAs function as signaling molecules, either enhancing or suppressing transcription processes, thus playing a key role in gene regulation. Given that lncRNAs post-transcriptionally regulate mRNAs, it is feasible to predict their interactions and investigate the associated mechanisms in pathological processes through regulatory networks [36]. A substantial body of research has highlighted the critical impact of lncRNAs in the development of NAFLD [37,38,39]. Additionally, numerous studies have found that the majority of lncRNAs accelerate or attenuate the progression of NAFLD primarily by sponging microRNAs, including *CCAT1*, *PVT1, lnc-SPARCL1-1:2*, *UC.372*, *MALAT1*, *UC.333*, and *NEAT1* [40,41,42,43,44]. Two studies reported that lncRNA *NEAT1* expression was elevated in both the serum and peripheral blood mononuclear cells (PBMCs) of patients with NAFLD. Additionally, the area under the curve for diagnosing NAFLD using NEAT1 levels in PBMCs was 0.822, showing a sensitivity of 86.47% and a specificity of 82.03% [44,45]. The experimental findings reported by Meng et al. [46] indicate that over-expression of lncRNA *MEG3* significantly diminished the levels of FOXO1, ACC1, and FAS, leading to a reduction in lipid accumulation within HepG2 cells. This study further demonstrated that lncRNA *MEG3* regulates de novo lipogenesis by reducing the expression and nuclear translocation of FOXO1 in HepG2 cells. These findings suggest that lncRNA *MEG3* might serve as a promising therapeutic target for lipid metabolic disorders. In our study, we identified several PM2.5-regulated lncRNA-mRNA-nets, providing a significant understanding of the mechanisms related to PM2.5 exposure and its effects on the pathogenesis of NAFLD and hepatic damage.

In the turquoise module, four hub lncRNAs (*Gm5848*, *Zfp141*, *Rmrp*, and *Rb1*) linked to hub mRNAs (*Psmb3*, *Mad211*, *Sdhd*, *Ndufa6*, *Snrqd2*, and *Immp11*) and are mainly related to NAFLD, multiple neurodegenerative diseases, oxidative phosphorylation, cell cycle, inflammation, and metabolic pathways. Yin et al. [47] investigated the function of lncRNA *RMRP* in modulating NAFLD. In both liver tissues from NAFLD patients and rat models, it was observed that *RMRP* showed increased expression, while *miR-206* exhibited reduced expression. *RMRP* inhibition improved the pathological condition and liver function indices associated with lipid accumulation in the hepatic tissues of rats with NAFLD while alleviating steatosis and reducing triglyceride secretion in AML-12 cells treated with free fatty acids (FFAs). Furthermore, *RMRP* inhibition can attenuate lipid accumulation and prevent NAFLD progression by targeting the miR-206/PTPN1 axis and down-regulating the miR-206/PTPN1-SREBP1C and PTPN1-PP2A-SP1-SREBP1C signaling pathways in both rat models of NAFLD and FFA-treated AML-12 cells. Multiple independent studies have verified the cancer-promoting impact of lncRNA *RMRP* in HCC. Zhou et al. [48,49] found that in HCC cells and a xenografted tumor model in mice, *RMRP* silencing inhibited migration, cell proliferation, and invasive properties, induced cell cycle arrest, and attenuated the tumorigenesis process. These effects are mediated through the regulation of *miR-613* expression. Additionally, over-expression of *RMRP* has been observed in HCC patients, correlating with tumor aggressiveness and poor clinical outcomes. Zhao et al. [50] showed that *RMRP* plays a role in the sequestration of *miR-206* and the activation of the TACR1/Erk1/2 pathway. Consequently, over-expression of *RMRP* has been identified as a prognostic marker associated with adverse outcomes in patients. However, Shao et al. [51] demonstrated down-regulation of *RMRP* in HCC cells, HCC patients, and xenografted tumor models in rats. Enforced over-expression of *RMRP* in these cells increased the apoptosis rate by modulating *miR-766* expression. Furthermore, following the silencing of *RMRP*, the expression profiles of multiple miRNAs (including *miR-206*, *miR-613*, *miR-1-3p*, and *miR-217*) were altered, indicating the extensive influence of the *RMRP*-miRNA regulatory network [49]. Anwar et al. [52] conducted a systematic screen to identify imprinted genes deregulated in human HCCs and found that *Rb1* exhibits imprinting anomalies, characterized by frequent hypermethylation or hypomethylation within the CpG island located in intron2 of *Rb1*. Chand et al. [53] found that *Rb1* is functionally deactivated in HCC via phosphorylation mediated by CYCLIN D/E. Additionally, human HCCs depend on the FOXM1-FOXO1 axis to facilitate the phosphorylation and inactivation of *Rb1*, thereby evading *Rb1*-induced senescence. Therefore, we postulated that hub lncRNAs, including *RMRP* and *Rb1*, which are co-expressed with several hub mRNAs in our WGCNA analysis, might contribute to PM2.5-induced NAFLD through the miR-206/PTPN1 axis, the miR-206/PTPN1-SREBP1C and PTPN1-PP2A-SP1-SREBP1C signaling pathways, metabolic processes, and phosphorylation. Additionally, both *RMRP* and *Rb1* have been shown to induce hepatocyte injury and contribute to the progress of HCC through mechanisms involving cell senescence, apoptosis, and the regulation of the cell cycle.

In the blue module, three hub lncRNAs (*Gm15446*, *Adh6-ps1*, and *Tmem181b-ps*) were found to interact with hub mRNAs (*Tsc2*, *Baat*, *Adipor2*, *Fpgt*, *Keg1*, and *Alg6*), and are primarily related to NAFLD, cell senescence, apoptosis, metabolic pathways, as well as p53, PI3K-AKT, and WNT signaling pathways. *Adh6-ps1* is a liver-expressed pseudogene in mice and belongs to the alcohol dehydrogenase gene complex (Adh). Its two primary miRNA targets, *Mir455* and *Mir511*, have been implicated in HCC when silenced [54]. Nicholson et al. [55] examined RNA alterations within the brain of a mouse model of multiple system atrophy, a rare neurodegenerative disorder. Using RNA sequencing, they analyzed brain samples from a well-established PLP-α-synuclein transgenic mouse model and identified 40 DEGs. Notably, the lncRNA *Gm15446* was recognized as one of the most prominent DEGs in the PLP mouse model dataset. Wang et al. [56] identified *Tsc2* as a candidate gene associated with a quantitative trait locus linked to hepatic steatosis and further investigated its role in regulating de novo lipogenesis. They found that *Tsc2* directly regulates lipogenesis in vivo by up-regulating the expression of lipogenic genes, such as *Fasn* and *Elovl6*. Midorikawa et al. [57] analyzed 52 early-stage and 108 overt HCC specimens using genome sequencing and found that mutations in the p53/RB1, PI3K-AKT, and WNT pathways are frequently observed in HCC. Consequently, based on our WGCNA analysis, we predicted that hub lncRNAs, including *Gm15446*, *Adh6-ps1*, and *Tmem181b-ps*, which were co-expressed with hub mRNAs such as *Tsc2* might contribute to PM2.5-induced NAFLD and its progression to HCC by regulating miRNAs such as *miR-455* and *miR-511*, lipogenesis, as well as p53/RB1, PI3K- AKT, and WNT pathways.

Moreover, in a mouse model, Dong et al. [58] demonstrated that acetyl-CoA carboxylase 1 (*ACACA*) as a key mRNA, plays an essential role in fatty acid biosynthesis. Furthermore, they showed that inhibiting *ACACA* reduces lipid accumulation in a hepatocyte model of fatty acid excess. This finding is consistent with our results. Specifically, in the brown modules of our WGCNA analysis, we identified that *ACACA* (an mRNA), which is involved in metabolic pathways, fatty acid biosynthesis, fatty acid metabolism, and the AMPK signaling pathways, is modulated by the lncRNA *Gm9958*. Consequently, our study suggests that *Gm9958* (a lncRNA) may serve as a promising therapeutic target for NAFLD.

## 4. Materials and Methods

### 4.1. Data Retrieving and Processing

The gene expression analysis for GSE186900, which relied on the Affymetrix GeneChip mouse Genome 430 2.0 array (Affymetrix, Inc., Santa Clara, CA, USA), was retrieved from the GEO repository (https://www.ncbi.nlm.nih.gov/geo/query/acc.cgi?acc=GSE186900, accessed on 10 December 2024). The GSE186900 dataset comprised ten liver tissue samples: five normal tissues and five tissues exposed to PM2.5. HISAT2 and StringTie were employed to realign the reads to the human reference genome along with its gene annotations [59].

### 4.2. Analysis of Differentially Expressed Genes

We used the “limma” package in R software (Version 4.1.1) to identify differentially expressed genes (DEGs). The “limma” package employs an F-statistic to detect DEGs across multiple groups. Empirical Bayes moderation was used to adjust the *p*-values. We used the Benjamini–Hochberg procedure for multiple testing correction and employed the false discovery rate (FDR) to correct *p*-values. Significance thresholds were set at *p* < 0.05, FC > 1.2, and FDR < 0.05 to identify significant differences in lncRNA and mRNA expression.

### 4.3. GO and Pathway Enrichment Analysis of DEGs

Gene Ontology (GO) provides a globally standardized method to categorize gene functions. It offers a flexible and comprehensive vocabulary for detailing the characteristics of genes and their products in living organisms. Following the GO annotation of DEGs, enrichment analysis was used to identify their core biological roles. Pathway analysis further enhances the understanding of gene-related biological functions. The Kyoto Encyclopedia of Genes and Genomes (KEGG) database serves as a primary public resource for pathway information. Pathway enrichment analysis uses KEGG pathways as units and applies a hypergeometric model to detect pathways with significant DEG representation relative to the genome [60]. Statistical significance was evaluated via Fisher’s exact test, with multiple comparison corrections calculating adjusted *p*-values and FDR. Differences were considered statistically significant at *p* < 0.05 and FDR < 0.05.

### 4.4. Weighted Gene Co-Expression Network Analysis (WGCNA)

WGCNA identifies correlated gene expression clusters and links them to disease phenotypes [61]. We conducted WGCNA on all genes using the “WGCNA” package in R software (Version 4.1.1). To ensure the robustness and reliability of the statistical results, we selected a subset of 58 lncRNAs and 3945 mRNAs for WGCNA analysis. Additionally, we assessed the similarity in expression patterns between pairs of genes by calculating the Pearson correlation coefficient. The β value represents the soft threshold power that balances independence with overall connectivity within co-expression modules. Based on the chosen lncRNAs and mRNAs, we set β = 20. Ultimately, we constructed a hierarchical clustering dendrogram of genes based on the Pearson correlation coefficient. Different branches and colors in the clustering tree represent unique gene modules. Modules were merged according to the Pearson correlation coefficient for further analysis [62].

### 4.5. Functional Enrichment Analysis

The GO database was utilized to analyze the biological process and molecular function within the mRNA modules. Additionally, KEGG pathway annotations were used to identify the key mRNAs and their roles in signaling pathways within the modules [60]. Consequently, we predicted and analyzed the essential mRNAs and associated signaling pathways using the functional enrichment analysis of Go and KEGG pathways.

### 4.6. Construction of lncRNA-mRNA-Nets and lncRNA-mRNA-Pathway-Nets

Hub genes are defined as genes that exhibit greater module connectivity. Highly connected hub genes acted as upstream regulators, whereas those with lower connectivity served as downstream regulators. Following the identification of the hub genes, we quantified the strength of co-expression interactions and examined the regulatory interactions between lncRNA-mRNA and mRNA-mRNA pairs to construct the lncRNA-mRNA-nets. Subsequently, the lncRNA-mRNA-nets and the key mRNA-regulated signaling pathways were integrated to form the lncRNA-mRNA-pathway-nets. The pathway network could clarify the signaling pathways in lncRNA regulation and predict their mechanisms in NAFLD.

### 4.7. Construction of Protein–Protein Interaction (PPI) Network

The PPI network analysis utilized the STRING (version 11.5; https://cn.string-db.org/, accessed on 17 January 2025) [63]. In our study, STRING was employed to analyze the interactions between lncRNAs and mRNAs among the identified modules.

### 4.8. Statistical Analysis

Statistical analyses were performed using R software (version 4.1.1) and SPSS (version 21.0). Student’s *t*-test and one-way ANOVA were employed for the statistical analysis of normalized data across different groups. A *p*-value< 0.05 was regarded as statistically significant.

## 5. Conclusions

Using WGCNA, we identified hub lncRNAs and mRNAs associated with NAFLD related to PM2.5 and constructed both lncRNA-mRNA-nets as well as lncRNA-mRNA-pathway-nets. Our findings demonstrated that hub lncRNAs could be involved in PM2.5-induced NAFLD by modulating cell senescence and apoptosis, metabolic pathways, fatty acid biosynthesis, inflammatory responses, and oxidative stress. Consequently, these identified hub lncRNAs may serve as potential biomarkers for NAFLD induced by PM2.5. Furthermore, WGCNA analysis identified its promising role as a novel predictive method for PM2.5-linked NAFLD. Therefore, future research should investigate the effects of hub lncRNA-mRNA interactions to facilitate the development of innovative and effective biomarkers for NAFLD diagnosis.

## Figures and Tables

**Table 1:** The number of lncRNAs and mRNAs across the 17 modules.

Module	Number of All	Number of mRNAs	Number of lncRNAs
Turquoise	1399	1379	20
Blue	1004	995	9
Brown	355	346	9
Yellow	175	170	5
Green	147	146	1
Red	109	108	1
Black	95	92	3
Pink	95	94	1
Magenta	93	91	2
Purple	88	87	1
Greenyellow	79	77	2
Tan	73	73	0
Cyan	69	68	1
Salmon	69	67	2
Midnight blue	68	68	0
Light cyan	63	63	0
Grey	4	3	1

**Table 2:** The top 15 significantly changed GOs of DEGs in blue and turquoise modules.

Module	Go Id	Go Name	Enrichment	*p*-Value
blue module	GO:0006357	regulation of transcription by RNA polymerase II	4.604618417	4.0808 × 10^−33^
	GO:0000122	negative regulation of transcription by RNA polymerase II	5.469731618	1.36261 × 10^−27^
	GO:0045944	positive regulation of transcription by RNA polymerase II	4.766936394	1.36552 × 10^−27^
	GO:0016310	phosphorylation	6.49228687	4.00222 × 10^−25^
	GO:0006974	cellular response to DNA damage stimulus	6.62509856	1.2414 × 10^−21^
	GO:0007165	signal transduction	3.841899685	6.64258 × 10^−18^
	GO:0006355	regulation of transcription, DNA-templated	3.92919919	3.5163 × 10^−17^
	GO:0006629	lipid metabolic process	5.406814483	1.19225 × 10^−16^
	GO:0045893	positive regulation of transcription, DNA-templated	4.949471573	4.01724 × 10^−16^
	GO:0006468	protein phosphorylation	5.130762335	6.86727 × 10^−16^
	GO:0006325	chromatin organization	6.945588197	2.18748 × 10^−15^
	GO:0015031	protein transport	4.820964444	2.30318 × 10^−15^
	GO:0007030	Golgi organization	12.96249113	8.20478 × 10^−14^
	GO:0006281	DNA repair	5.872692459	1.71088 × 10^−13^
	GO:0043066	negative regulation of the apoptotic process	4.746593849	2.40923 × 10^−13^
turquoise module	GO:0015031	protein transport	5.64659069	4.72814 × 10^−28^
	GO:0006357	regulation of transcription by RNA polymerase II	3.62721593	2.76769 × 10^−27^
	GO:0043066	negative regulation of the apoptotic process	5.658760066	4.18637 × 10^−25^
	GO:0006397	mRNA processing	6.342185195	1.2486 × 10^−19^
	GO:0007049	cell cycle	4.580467085	7.40711 × 10^−19^
	GO:0006412	translation	6.837668413	2.47991 × 10^−17^
	GO:0006355	regulation of transcription, DNA-templated	3.287217115	9.34773 × 10^−16^
	GO:0032981	mitochondrial respiratory chain complex I assembly	17.86166443	1.08356 × 10^−15^
	GO:0008380	RNA splicing	6.472296819	1.18584 × 10^−15^
	GO:0000122	negative regulation of transcription by RNA polymerase II	3.449634515	9.34622 × 10^−15^
	GO:0045944	positive regulation of transcription by RNA polymerase II	3.065161702	1.4967 × 10^−14^
	GO:0006974	cellular response to DNA damage stimulus	4.398665041	8.9227 × 10^−14^
	GO:0043161	proteasome-mediated ubiquitin-dependent protein catabolic process	7.501899059	9.65769 × 10^−14^
	GO:0006511	ubiquitin-dependent protein catabolic process	5.86085864	1.08627 × 10^−12^
	GO:0016567	protein ubiquitination	5.144327333	1.63207 × 10^−12^

**Table 3:** The top 15 significantly altered pathways of DEGs in blue and turquoise modules.

Module	Pathway Id	Pathway Name	Enrichment	*p*-Value
blue module	01100	Metabolic pathways	4.373215837	2.05556 × 10^−31^
	05200	Pathways in cancer	4.81652525	1.57133 × 10^−13^
	05163	Human cytomegalovirus infection	7.005463957	5.87512 × 10^−13^
	05168	Herpes simplex virus 1 infection	4.894647306	6.88501 × 10^−12^
	04144	Endocytosis	6.318653765	1.60792 × 10^−11^
	04152	AMPK signaling pathway	9.488882397	6.80369 × 10^−11^
	05165	Human papillomavirus infection	4.954140257	8.83013 × 10^−10^
	05205	Proteoglycans in cancer	6.561215023	1.8569 × 10^−9^
	04925	Aldosterone synthesis and secretion	9.523767994	5.10304 × 10^−9^
	04142	Lysosomes	7.985872399	1.24271 × 10^−8^
	05152	Tuberculosis	6.642217678	1.4244 × 10^−8^
	04151	PI3K-Akt signaling pathway	4.579244778	2.09365 × 10^−8^
	04934	Cushing syndrome	6.918976748	2.50236 × 10^−8^
	05160	Hepatitis C	6.79317717	3.21535 × 10^−8^
	05166	Human T-cell leukemia virus 1 infection	5.445542833	3.60359 × 10^−8^
turquoise Module	01100	Metabolic pathways	5.115202324	8.71148 × 10^−59^
	05168	Herpes simplex virus 1 infection	6.568939087	1.82711 × 10^−27^
	05010	Alzheimer disease	6.670896013	6.91349 × 10^−23^
	04714	Thermogenesis	8.561950013	3.59115 × 10^−22^
	05012	Parkinson disease	8.194128949	4.42746 × 10^−22^
	05014	Amyotrophic lateral sclerosis	6.374411746	3.97572 × 10^−21^
	05022	Pathways of neurodegeneration—multiple diseases	5.446956194	3.79844 × 10^−20^
	05016	Huntington disease	6.882950985	6.23685 × 10^−20^
	05020	Prion disease	6.939723166	4.91892 × 10^−18^
	00190	Oxidative phosphorylation	10.28220814	1.25791 × 10^−17^
	04932	Non-alcoholic fatty liver disease	9.418775033	2.63225 × 10^−17^
	05200	Pathways in cancer	4.331782567	7.20715 × 10^−15^
	03040	Spliceosome	8.980818214	2.52467 × 10^−14^
	04141	Protein processing in endoplasmic reticulum	7.314715047	5.75875 × 10^−13^
	04630	JAK-STAT signaling pathway	5.826179003	1.07188 × 10^−8^

## Data Availability

The original data presented in the study are openly available in GEO datasets at: https://www.ncbi.nlm.nih.gov/geo/query/acc.cgi?acc=GSE186900 (accessed on 10 December 2024).

## Supplementary Materials

The following supporting information can be downloaded at: https://www.mdpi.com/article/10.3390/ijms26062808/s1.

## Abbreviations

The following abbreviations are used in this manuscript:

PM2.5	Fine particulate matter
WGCNA	Weighted gene co-expression network analysis
NAFLD	Non-alcoholic fatty liver disease
lncRNAs	Long noncoding RNAs
mRNAs	messenger RNAs
GEO	Gene Expression Omnibus
GO	Gene Ontology
KEGG	Kyoto Encyclopedia of Genes and Genomes
DEGs	Differentially expressed genes
HCC	Hepatocellular carcinoma
β	Soft threshold power
FDR	False discovery rate
FC	Fold change
PPI	Protein–protein interaction
OR	Odds ratio
HR	Hazard ratios
AED	Aerodynamic diameter
