# Supplementary material for: Identification and Assessment of lncRNAs and mRNAs in PM2.5-Induced Hepatic Steatosis
Source: Int J Mol Sci. 2025 Mar 20;26(6):2808. doi: 10.3390/ijms26062808 (PMC11943408; doi:10.3390/ijms26062808)
Supplement: Supplementary file 1 [file ijms-26-02808-s001.zip › ijms-3521388-supplementary.pdf]

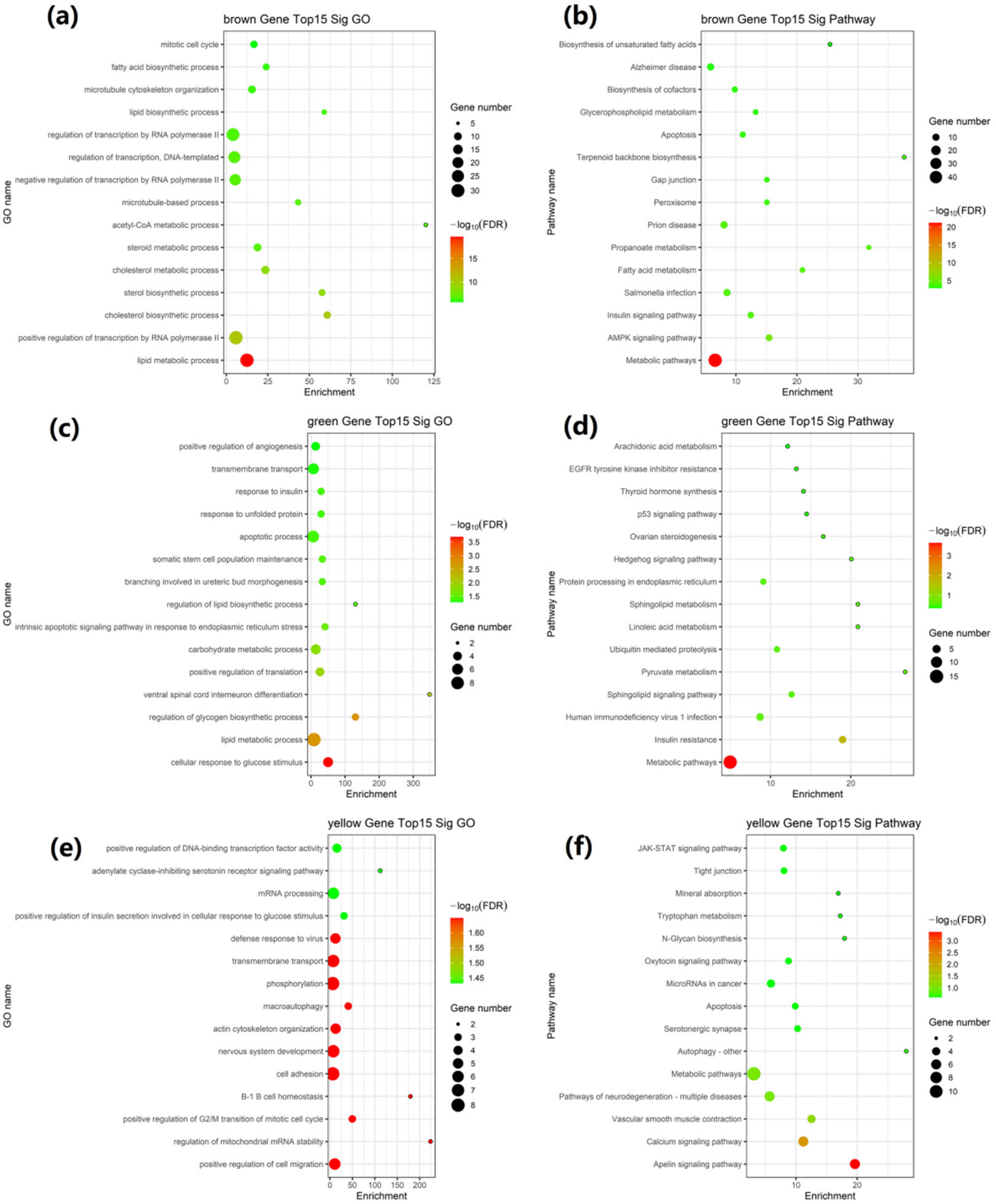

Figure S1. Functional enrichment analysis in the brown, green, and yellow modules. (a) (b) GO and KEGG Pathway enrichment analysis of mRNAs in the brown module; (c) (d) the green module; (e) (f) the yellow module.

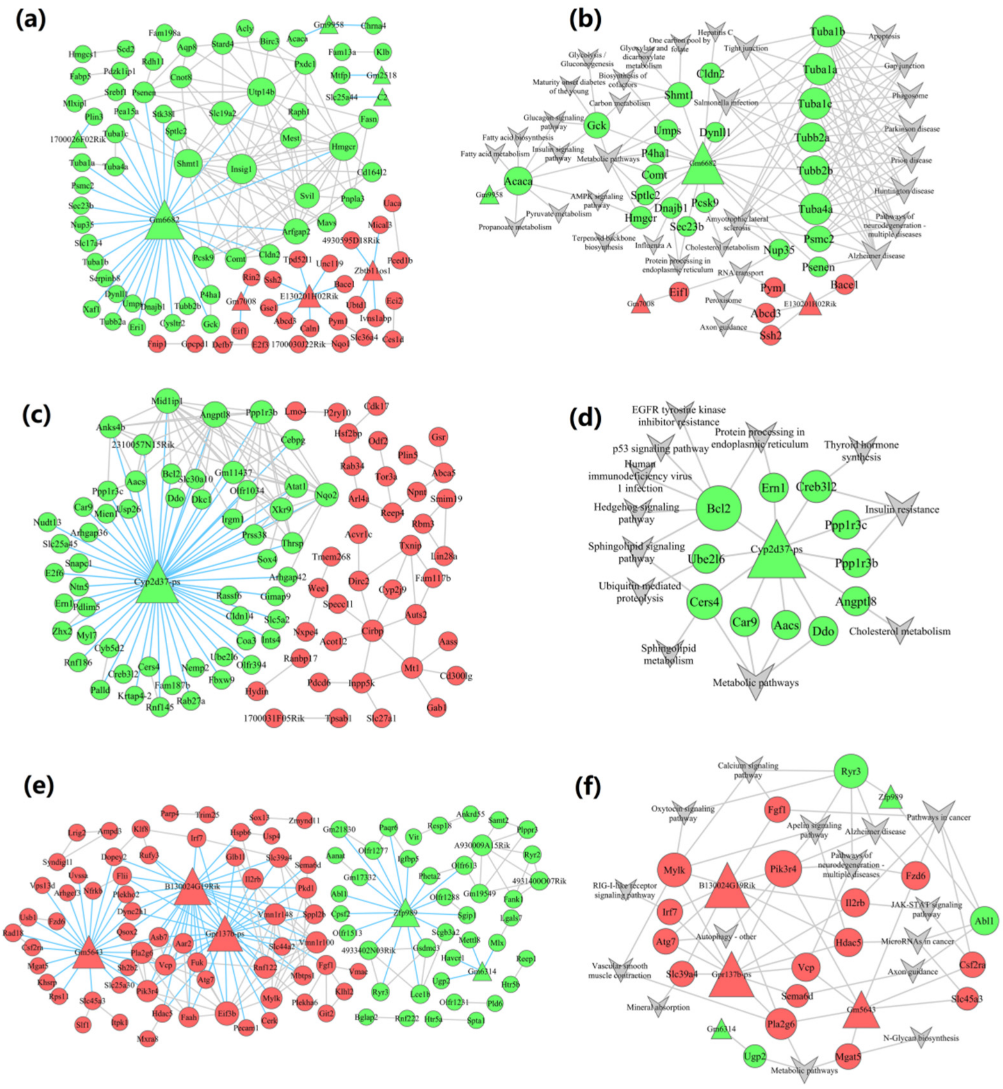

Figure S2. lncRNA-mRNA-nets and LncRNA-mRNA-pathway-nets of hub genes in the brown, green, and yellow modules. (a) (b) lncRNA-mRNA-nets and lncRNA-mRNA-pathway-nets of the brown module; (c) (d) the green module; (e) (f) the yellow module (Triangles represent lncRNAs, circles represent mRNAs, and red and green graphics represent up-regulated and down-regulated genes. The size of the circle represents the regulation capacity of the mRNA, with larger circles indicating relatively stronger regulation capacity.)
